# Removal of ecotoxicity of 17α-ethinylestradiol using TAML/peroxide water treatment

**DOI:** 10.1038/srep10511

**Published:** 2015-06-12

**Authors:** Matthew R. Mills, Karla Arias-Salazar, Alice Baynes, Longzhu Q. Shen, John Churchley, Nicola Beresford, Chakicherla Gayathri, Roberto R. Gil, Rakesh Kanda, Susan Jobling, Terrence J. Collins

**Affiliations:** 1 Institute for Green Science and Department of Chemistry, Carnegie Mellon University, 4400 Fifth Avenue, Pittsburgh PA 15213; 2 Institute of Environment, Health and Societies, Brunel University London, Uxbridge, Middlesex UB83PH, UK

## Abstract

17α-ethinylestradiol (EE2), a synthetic oestrogen in oral contraceptives, is one of many pharmaceuticals found in inland waterways worldwide as a result of human consumption and excretion into wastewater treatment systems. At low parts per trillion (ppt), EE2 induces feminisation of male fish, diminishing reproductive success and causing fish population collapse. Intended water quality standards for EE2 set a much needed global precedent. Ozone and activated carbon provide effective wastewater treatments, but their energy intensities and capital/operating costs are formidable barriers to adoption. Here we describe the technical and environmental performance of a fast- developing contender for mitigation of EE2 contamination of wastewater based upon small- molecule, full-functional peroxidase enzyme replicas called “TAML activators”. From neutral to basic pH, TAML activators with H_2_O_2_ efficiently degrade EE2 in pure lab water, municipal effluents and EE2-spiked synthetic urine. TAML/H_2_O_2_ treatment curtails estrogenicity *in vitro* and substantially diminishes fish feminization *in vivo*. Our results provide a starting point for a future process in which tens of thousands of tonnes of wastewater could be treated per kilogram of catalyst. We suggest TAML/H_2_O_2_ is a worthy candidate for exploration as an environmentally compatible, versatile, method for removing EE2 and other pharmaceuticals from municipal wastewaters.

Increasing evidence suggests that the presence of trace levels of pharmaceuticals in the environment can vitiate ecologies^1^. The most well researched effect involves the feminization of male fish exposed to effluents from wastewater treatment plants containing ethinylestradiol (EE2)^2^. EE2, a synthetic oestrogen in oral contraceptives, is found in inland waterways worldwide^3^ emanating from human excretion into wastewater treatment systems. At low parts per trillion (ppt), EE2 induces feminisation of wild male fish^4^, diminishing reproductive success^5^,^6^. Indeed in large-scale lake experiments in Canada^7^, 5–6 ppt EE2 led to the collapse of the lake’s population of fathead minnows, *Pimephales promelas*, and to longer term indirect effects on the lake foodweb^8^. Other examples of potential conflict between pharmaceutical use and ecological integrity include population collapse of Asian vultures contaminated by diclofenac^9^, and contamination of water and soil with antibiotics causing antimicrobial resistance^10^.

Within the EU, a combination of factors present a compelling case for mitigating pollution of rivers by EE2 and other pharmaceuticals^11^. These include: high human population density, increasing pharmaceutical consumption, intensifying animal husbandry, and limited water supplies. In practice, however, mitigation is hard to implement due to technical difficulties associated with monitoring these chemicals at low concentrations and the disproportionate cost of widespread installation of water treatment technologies able to remove them^12^,^13^.

Although considered one of the biggest public health accomplishments of the 20^th^ century, current wastewater treatment systems are not sustainable options for removing pharmaceuticals: Treatment by Activated Sludge Process (ASP) has proven to be inadequate at removing numerous pharmaceuticals creating the need for advanced treatment options. Ozone and activated carbon (AC) are the leading contenders for advanced treatment^11^ but their energy requirements, as well as capital and operating costs are formidable barriers to widespread adoption^12^, especially for smaller communities.

A technically effective, environmentally compatible and cost-effective way to overcome the impracticality of using ozone or AC for removing pharmaceuticals from wastewater may come from treatment with hydrogen peroxide catalysed by extremely efficient TAML catalysts. Iron TAML activators (**1**, **2**, Fig. 1) are full-functional, small molecule mimics of the peroxidase enzymes that at nanomolar concentrations (<100 nM) can decompose many micropollutants. For example, in laboratory studies, TAML/peroxide has been shown to efficiently degrade the persistent drug sertraline via its oxidative metabolites^16^, the recalcitrant pesticides trichlorophenol and pentachlorophenol^17^, thiophosphate pesticides such as fenitrothion^18^, dyes^19^,^20^ and many other micropollutants^21^. Preliminary studies on U.K municipal wastewater show that TAML/peroxide readily degrades numerous pharmaceuticals^14^.

The contraceptive pill hormone EE2 causes well-documented severe impacts on aquatic life^15^, therefore this study investigates the efficacy of TAML/peroxide at removing EE2 and it’s biological effects. Preliminary studies at high pH (8-10) and high concentrations of hydrogen peroxide (4 mM, 136 ppm) indicate that removal of EE2 and other estrogens (estradiol (E2), estrone (E1) all at 80 μM) with TAML (83 nM **1**) is possible^22^. In addition, *in vivo* toxicity tests using embryonic zebrafish suggest TAML is not overtly toxic to fish^23^ and *in vitro* tests also show TAML itself does not show inherent endocrine disrupting activity^24^. Taken together, these studies set the stage for the current multidisciplinary, cross-sector study to investigate the removal of EE2 from both laboratory waters and real wastewater, under more environmentally appropriate conditions. These include neutral pH, lower hydrogen peroxide concentrations and estrogen concentrations of more than a million-fold lower than previously tested, which are much more environmentally relevant and still ecotoxic to fish.

## Results

### Degradation at various pH with TAML activators 1 and 2

**Figure 2:**
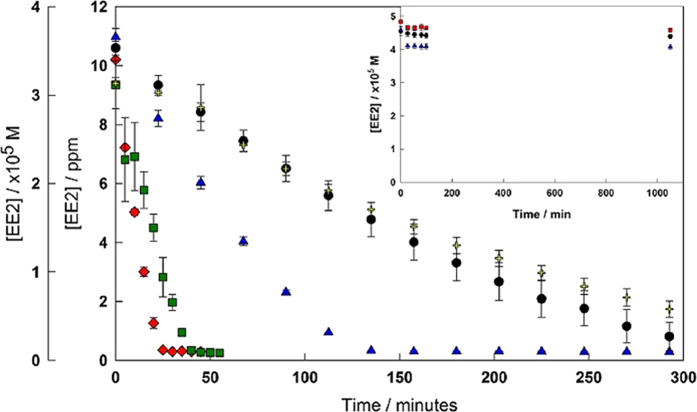
Degradation of EE2 under a variety of environmentally relevant conditions. *Graph:* Degradation of EE2 at pH 7.5 (phosphate buffer, 0.01 M) with H_2_O_2_ (337 μM, 11.46 ppm) catalysed by **2** (10 nM, black circles), (25 nM, blue triangles), (34 nM, green squares), (80 nM, red diamonds) and catalysed by **1** (80 nM, yellow crosses). *Inset:* Concentration of EE2 with no catalyst and 50 equivalents of hydrogen peroxide. Conditions: [EE2] = 5×10^-5^ M (14 ppm), [H_2_O_2_] = 2.5×10^-3^ M (85 ppm), 0.01 M phosphate buffer at pH 6 (black circles), pH 7.5 (red squares), and pH 9 (blue triangles).

Analogue **2** a more aggressive form of the parent TAML activator **1** (Fig. 2), are emphasized due to its superior performance over a pH range typically found in wastewaters. With this catalyst, degradation of EE2 (~10 ppm) with a fixed H_2_O_2_ concentration (11.46 ppm) at pH 7.5 was completed within 25 min with 80 nM catalyst. The peroxide concentration was chosen in order to remain in excess of EE2. At this concentration, the peroxidase-like behaviour of TAML catalysts greatly outweighs any catalase-like behaviour^25^. The degradation time increased to 5 hours at 10 nM (5.4 ppb; Fig. 2). Taken together, the results show that **2** is about 10 times more effective than **1** at pH 7.5.

### Determination of rate constant *k*_II_

The EE2 oxidation rate constant with **1** was experimentally determined to be 8.6 ± 0.5 ×10^5^ M^-1^ s^-1^ (Supplementary information Fig. S2). With this information, the rate of degradation of EE2 in water at pH 7 could be reliably predicted (Supplementary information Eq. S1). At pH 7, the activation of the catalyst, described by rate constant *k*_I_, is usually the rate-determining step. However, at the extremely low concentrations of EE2 found in wastewater effluent and environmental waters, the oxidation of EE2 by the active catalyst will be the rate-determining step as long as the concentration of hydrogen peroxide is greater than approximately 100 ppb (3 μM). When compared to rate constants of other oxidants at pH 7, which have been measured by other researchers, the *k*_II_ value is higher than ferrate, chlorite, hypochlorite, hypobromite and monochloramine, comparable to ozone and exceeded by hydroxyl radicals^26^,^27^.

### Degradation of EE2 to isolate reaction intermediates

**Figure 3:**
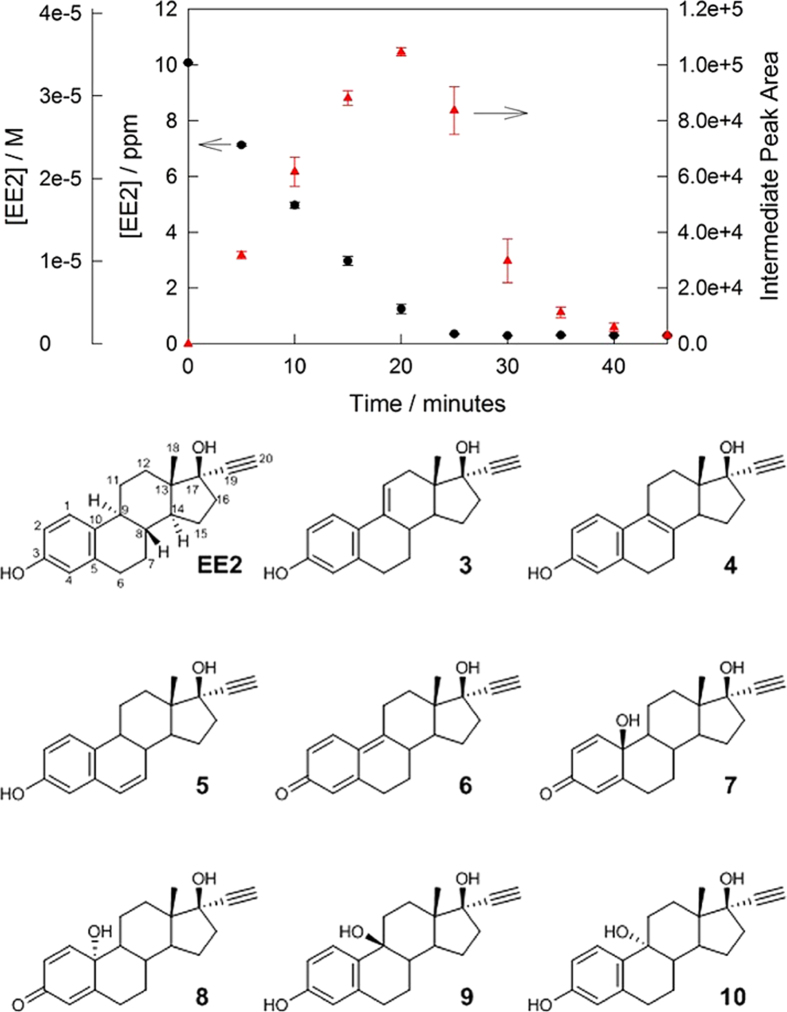
Typical degradation of EE2 with formation and subsequent degradation of intermediates. *Top:* Decay of EE2 (10 ppm) with H_2_O_2_ (337 μM, 11.46 ppm) catalysed by **2** (80 nM, black) showing the combined formation and decay of three early degradation intermediates (red); pH 7.5 (phosphate buffer, 0.01 M). *Bottom*: Identified and likely EE2 degradation intermediates (also see Supplementary information).

HPLC investigation of EE2 degradation with **1** or **2** and peroxide revealed the early formation and disappearance of two peaks with shorter retention times than EE2—the combined peak area attained a maximum as EE2 concentration reached its minimum, then declined rapidly and completely (Fig. 3; Top). UV spectra of the material in the new peaks are similar to the UV spectrum of EE2 suggesting early degradation intermediates. After isolation and purification *via* preparative HPLC, ESI-MS analysis revealed two masses common to the constituents of both HPLC peaks, 294 m/z (EE2–H_2_) and 312 m/z (EE2 + O)—a set of plausible structures based on known chemistry is shown in Fig. 3; Bottom. NMR studies of the two isolated fractions revealed that both consisted of mixtures of four compounds.

By comparing the ^1^H NMR spectrum of the intermediate mixture with the measured spectra of purchased **3**, **4** and **5** and the reported data for **7**/**8** (produced via a screening TAML/peroxide EE2 oxidation)^28^, structural assignments were made. While ∆-9(11)-EE2 **3** was present in the mixture, ∆-8(9)-EE2 **4** and ∆-6(7)-EE2 **5** were not. Further analysis of the ^1^H and ^13^C and 2D NMR spectra (Supplementary information Figs. S4-S7) indicated a high probability that **8**, **9** and **10** comprise the remaining members of the product mixture. A possible explanation for the HPLC and NMR data is that an initial oxidation intermediate forms which adds H_2_O under the catalytic conditions to give the multiple observed alcohol oxidation products. Compound **6** is primed for such water additions under catalytic conditions: an analogous product of electrochemical oxidation of E2 has been proposed^29^.

### Determination of estrogenic activity of reaction intermediates *in vitro* via Yeast Estrogen Screen (YES) assay

The three possible intermediates available for purchase (**3**, **4** and **5**) were tested in the Yeast Estrogen Screen (YES), a reporter gene assay which incorporates a recombinant human estrogen receptor^30^. All three compounds elicited estrogenic activity *in vitro* in the YES assay. In this assay **5** (∆-6(7)-EE2) was 57% as potent as EE2, **4** (∆-8(9)-EE2) was 52% as potent and **3** (∆-9(11)-EE2) was 35% as potent as EE2. As stated above, from the purchasable intermediates, only **3** was actually present in the intermediate mixture, and currently and **8**, **9** and **10** cannot be bought and therefore tested for estrogenic activity.

### Degradation of EE2 and removal of estrogenic activity *in vitro* - Yeast Estrogen Screen (YES) assays

To study the total degradation of EE2 via the intermediates at low, environmentally relevant concentrations, loss of estrogenicity with TAML/H_2_O_2_ treatment was measured using the YES assay (LOD = 0.1 ng.L-1 or ppt)^30^. Total estrogenicity (E2 equivalence; ppt EEQ) was completely removed by all treatments except the most mild ([1] = 80 nM and 0.16 ppm H_2_O_2_, unbuffered water, Supplementary information Fig. S1), indicating total destruction of all estrogenic intermediates in the higher H_2_O_2_ concentration experiments. These results emphasize the significant advantage of combining biological and chemical analysis as exhibited in this collaborative study. Mere destruction of the target compound, EE2, does not guarantee the removal of estrogenic activity or ecotoxicity.

### Degradation of EE2 in synthetic urine under controlled pH conditions

TAML/peroxide is also efficacious in removing EE2 from spiked synthetic urine. At pH 10, 100 nM **1** was able to completely degrade EE2 (10 μM) in synthetic urine with 60 mM H_2_O_2_ in 60 minutes. At pH 9, under the same conditions there was approximately 30% degradation in the same time frame (results not shown).

### Degradation of EE2 and removal of estrogenic activity *in vivo* – vitellogenin (VTG) induction in male fathead minnows.

**Figure 4:**
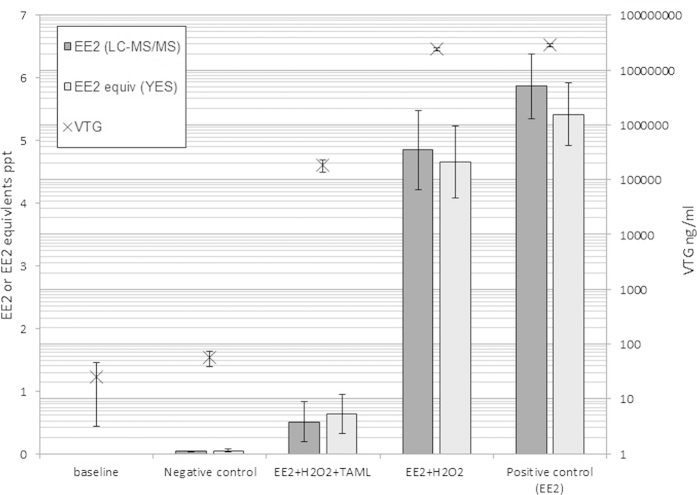
Average EE2 concentration and estrogenic activity in treated and untreated waters with plasma vitellogenin in male fish exposed to the treatments for 21 days. EE2 concentration (ng/l, ppt; dark grey bar, 1^st^ Y-axis) was measured by LC-MS/MS, estrogenic activity (EE2 equivalent ng/l, ppt; light grey bar, 1^st^ Y-axis) was measured via *in vitro* Yeast Estrogen Screen (YES). Plasma vitellogenin (ng/ml or ppb; grey cross X, 2^nd^ Y-axis log scale) concentration in male fathead minnows were measured via a quantitative enzyme-linked immunosorbent assay (ELISA). EE2 chemical analysis results reported as <0.03 ppt EE2 (i.e. lower than detection limit (LOD)) were treated as having half LOD (i.e. 0.015 ppt EE2) for use in calculations of averages, standard error and statistical analysis. EE2 and estrogenic activity are average measured concentrations sampled over the 21 day exposure. Plasma VTG was measured prior to exposure (baseline) and after 21 days exposure. The treatment regime consisted of; Control (water only), **2**/H_2_O_2_ + EE2, H_2_O_2_ + EE2, and EE2-only. All error bars represent standard error of the mean in all cases.

We next examined the ability of **2**/H_2_O_2_ to protect male fish from exposures to pharmaceutical estrogen by exposing fathead minnows to EE2 (nominally 6.8 pM (2ppt)) plus **2**/H_2_O_2_ ([**2**] 80 nM, [H_2_O_2_] = 4.7 μM (0.16 ppm)) treatment in continuous flow-through tanks. Treatment resulted in significantly lower (but still detectable) concentrations of EE2 in the EE2 + **2**/H_2_O_2_ tanks (0.5 ± 0.32 ppt, mean ± standard error) compared with the EE2-only (5.87 ± 0.51 ppt) and EE2 + H_2_O_2_ (4.85 ± 0.63 ppt) tanks (P = 0.004 and 0.006 respectively; ANOVA/LSD) (Fig. 4). This translates to 89.7% and 91.5% removal of EE2 in the EE2 + **2**/H_2_O_2_ tanks compared to the EE2 + H_2_O_2_ and EE2-only tanks. For all treatments, there was strong agreement between the analytical chemistry and *in vitro* YES assay results (Fig. 4) indicating all possible estrogenic intermediates had also been destroyed. Significantly lower levels of the vitellogenin (VTG) biomarker were also observed in the exposed male fish (Fig. 4) in the EE2 + **2**/H_2_O_2_ tanks compared to the EE2 + H_2_O_2_ and EE2-only tanks (P < 0.001 in both cases; ANOVA/LSD). Due to this biomarker’s extreme sensitivity to exogenous estrogens, the **2**/H_2_O_2_ + EE2 treatment fish still had elevated (although markedly reduced) VTG compared to the baseline (pre-exposure) and Control (no exposure) fish. The continuous flow-through set up and the low (non-toxic) concentration of H_2_O_2_ used in the *in vivo* study compared to the other experiments resulted in the very low residual concentration of EE2 in the EE2 + **2**/H_2_O_2_ treatment. No significant effects on fish mortality of the functioning **2**/H_2_O_2_ *in vivo* treatment (3% (2 fish) over 21 day experiment) or condition factor (1.44 ± 0.02) were observed. In a wastewater treatment facility longer retention time and higher H_2_O_2_ concentrations could be tested to reduce EE2 even further.

### Degradation of estrogens in Activated Sludge Process (ASP) effluent

**Figure 5:**
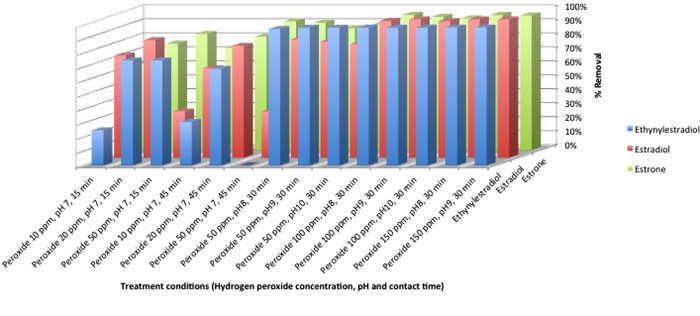
Effect of hydrogen peroxide concentration, pH and contact time on removal of steroid estrogens from sewage effluent using 1/H_2_O_2_. Conditions: [**1**] = 40 nM, room temperature, for pH 8, 9 and 10 reactions sodium hydroxide was added to the effluent to achieve desired pH.

Exploratory studies were performed to probe the efficacy of TAML/H_2_O_2_ (**1**, 40 nM) in removing EE2 from the effluent of a nitrifying activated sludge process (ASP) effluent at ambient temperature. In these limited laboratory studies, peroxide treatment in the presence of catalyst **1** was effective in reducing not only EE2, but also natural steroid estrogens, estrone (E1) and 17β-estradiol (E2). In general the removal of estrogens increased with increasing pH (pH 7, 8, 9), contact time (15, 30 and 45 min) and H_2_O_2_ concentration (10-150 ppm) (Fig. 5). The oxidative degradation of steroid estrogens in wastewater samples using TAML/H_2_O_2_ is dependent on a number of factors which include oxidant and catalyst dose, various water quality parameters such as organic and inorganic matter, dissolved and suspended solids as well as pH and temperature. At high pH, high removal of E1, E2 and EE2 occurs easily but at neutral pH or lower H_2_O_2_ dose the effectiveness of TAML/H_2_O_2_ treatment is reduced for EE2 compared to E1 and E2, due to the higher chemical stability of EE2.

In general the duplicate samples taken in each of the four experiments gave comparable results. The total iron data showed that the increase in iron with addition of the catalyst is very small, increasing by only 5 and 10 μg/l, a negligible amount from an environmental point of view.

Energy consumption is a major contributor to the operating cost of wastewater treatment plants and therefore an important parameter for choosing a treatment technology. As an example, ozone dose and therefore the energy consumption of ozonation is dependent on the characteristics of the wastewater to be treated and the type of chemical substances to be removed. A recent study on the energy consumption of ozone to treat estrogenic substances^31^ showed that between 0.14-0.90 kWh/m^3^ was required for the removal of a range of estrogenic compounds from water. For EE2 specifically, the energy required was 0.2 kWh/m^3^. Based on electricity costs of €0.16 kWh-1, it is estimated the cost of ozone treatment of EE2 would be 0.032 € m^3^ and with the inclusion of capital investment and other costs, ozone will typically have costs of €0.18 m^3^. Comparing this with the TAML/H_2_O_2_ process and assuming the cost of energy in the production of H_2_O_2_ is 10.81 kWh per Kg of H_2_O_2_^32^ then an application of 20 ppm would consume 0.216 kWh/m^3^ which is similar to 0.2 kWh of energy needed with ozone. A lower peroxide usage of 10 ppm would halve the energy consumption of the TAML process in comparison with ozone for EE2 removal, and would therefore be a cost and energy effective treatment. Therefore, cost and energy considerations favour a peroxide concentration of 10 ppm or lower. Process optimization is planned for pilot plant trials.

## Conclusion

Taken together, the studies presented here clearly illustrate that Fe-TAML catalysts are extremely effective in the removal of steroid estrogens in wastewater in ways that are likely to dramatically reduce the feminising effects of estrogenic sewage effluent discharges on wild fish in receiving waters. The application of Fe-TAML catalysts within wastewater treatment works is likely to represent a simple and effective environmentally sustainable alternative to current high technology advanced treatment processes.

## Materials and Methods

### Chemicals

All solvents were HPLC-grade unless otherwise stated. Analytical-grade EE2 and catalase (from *Aspergillus niger*, 2350 U mg^-1^) were obtained from Sigma-Aldrich. Hydrogen peroxide (30% w/w) was purchased from Fluka. The –H_2_ EE2 derivatives **3**, **4** and **5** were purchased from Dalton Pharma Services (Toronto, ON). All other reagents and solvents (at least ACS reagent grade) were obtained from commercial sources (Aldrich, Fisher, Acros, and Fluka). TAML activator FeB* (**1**) was synthesized following the published method^22^ and FeNO_2_BF_2_ (**2**) was obtained from GreenOx Catalysts (Pittsburgh, PA). Hydrogen peroxide stock solutions were standardized daily spectrophotometrically (ε = 72.4 M^-1^ cm^-1^)^23^. Phosphate buffer (0.1 M, potassium phosphate) was used as the reaction medium. The pH of each reaction mixture was adjusted by addition of potassium hydroxide or phosphoric acid solutions. Stock solutions of EE2 were prepared in methanol or ethanol (no effect from the alcohol was observed in the degradation pathway or rate) and were stored under refrigeration. Stock solution of **1** and **2** were prepared in methanol and were stored under refrigeration. Stock solutions of catalase (~23,500 U mL^-1^), used to quench the reactions by decomposing excess H_2_O_2_, were prepared in water and stored under refrigeration for a maximum of one week before disposal.

### Instrumentation and Analysis

A Shimadzu HPLC system [Shimadzu CMB-20A controller, LC-20AB pump, DGU-20A_3_ degasser, SPD-M20A diode array detector, RF-20A XS fluorimeter detector, CTO-20A column oven, and SIL-20A HT auto sampler] was used for monitoring the oxidative degradation of EE2. Chromatographic separation of EE2 and its degradation intermediates was achieved using an Agilent Microsorb-MV 100-5 C18 (250 mm x 4.6 mm, 5 μm) column. HPLC analysis conditions were: 25-uL injection volume, 40 °C column temperature, and isocratic elution using 40% acetonitrile and 60% water at 1-mL min^-1^ flow rate. The HPLC diode array detector was set to a 200–450 nm range and the fluorimeter detector was set to λ_ex_ = 220 and λ_em_ = 305 nm. The retention times of EE2 and its estrogenic degradation intermediates under these conditions were, respectively, 5.2, 3.0, and 3.4 minutes. ESI-MS analyses were performed using a Finnigan LCQ MS ion trap with ESI detection. A Bruker 500 MHz NMR instrument was used for ^1^H and ^13^C NMR studies (1D and 2D) at 300 K. The pH measurements were acquired with a Corning 220 pH meter calibrated with standard buffer solutions at pHs 4, 7, and 10.

### Degradation Comparison at varying pH

A volume of EE2 stock solution was added to an aqueous solution of phosphate buffer (0.01 M) at the desired pH (pH 6.0, 7.5 and 9.0) and was sonicated at 40 °C for 20 minutes to ensure a homogenous solution, then cooled to room temperature. To this solution, a volume of catalyst stock (**1** or **2)** solution was added to achieve the 80 nM. An aliquot of the reaction mixture was analyzed by HPLC to measure the initial concentration. To the remaining mixture, a volume of H_2_O_2_ was added to initiate the reaction. The concentration of EE2 was monitored by HPLC various time intervals (e.g. pH 7.5 - Fig. 2), either by direct injection of the reaction mixture into the instrument (1, pH 6 and 7.5), or by quenching 1 mL aliquots with a small volume of catalase solution in an HPLC vial. The concentrations of each component in the reaction mixture are as follows: [EE2] = 33.7 μM (10.00 ppm), [1] = 80.0 nM (0.04 ppm), [H_2_O_2_] = 337.4 μM (11.48 ppm).

### Determination of k_II_ at pH 7

*k*_II_ was determined by measuring the initial rate of degradation of EE2 at varying concentrations of EE2. The data was then plotted in a modified Lineweaver-Burke plot to linearize the data, with the slope of the line being equal to 1/*k*_II_ (Supplementary information Fig. S2). The value of *k*_II_ determined in this was 8.6 ± 0.5 ×10^5^ M^-1^ s^-^1. To verify this value, EE2 was subjected to catalyzed degradation under conditions in which *k*_II_ would be rate-limiting. This resulted in an exponential decay which was linearized by plotting ln(a_0_/a_t_) vs. time (Supplementary information Fig. S3). The slope of the line was equal to *k*_II_[Fe]. This resulted in a rate 9.4 ± 0.2 ×10^5^ M^-1^ s^-1^, which is within error of the previously calculated value.

### Degradation of EE2 to isolate reaction intermediates

An appropriate volume of EE2 stock solution was added to a 1L aqueous solution of pH 9.0 phosphate buffer (0.01 M) and was first sonicated at 40 °C for 20 minutes to ensure a homogenous solution, then was cooled to room temperature. To this solution, an appropriate volume of **1** stock solution was added. Degradation was then initiated by addition of an appropriate volume of H_2_O_2_ stock solution. At the experimentally determined time of maximum intermediate concentration, 1 mL of catalase was added to quench the remaining H_2_O_2_ in the reaction mixture. The concentrations of each component in the reaction mixture are as follows: [EE2] = 33.7 μM (10.00 ppm), [**1**] = 80.0 nM (0.04 ppm), [H_2_O_2_] = 337.4 μM (11.48 ppm). Sample was concentrated using solid phase extraction (Phenomenex Strata-X, 12 mL Gigatubes with 1g sorbent). SPE cartridges were eluted with acetonitrile and concentrated to 500 μL for purification with preparative HPLC.

### Determination of estrogenic activity of reaction intermediates *in vitro* via Yeast Estrogen Screen (YES) assay

EE2 reaction intermediates **3** (10.03 ppm), **4** (9.70 ppm) and **5** (9.74 ppm) (Dalton Pharma Services) and EE2 (10.00 ppm) were prepared as stock solutions in ethanol and assessed for estrogenic activity in the YES assay^30^. The other intermediate compounds were not tested by YES assay due to the inability to purchase them as standards.

### Degradation of EE2 and removal of estrogenic activity *in vitro* - Yeast Estrogen Screen (YES) assay

Experiments were conducted to determine if estrogenic activity (of the parent and intermediate compounds) had been completely removed after treatment or if activity still persisted. These experiments were also designed to inform the subsequent *in vivo* experiment which would need to use much lower (more environmentally relevant) concentration of EE2 and non-toxic concentrations of H_2_O_2_. Twelve reactions were conducted using three concentrations of H_2_O_2_ at either unbuffered or fixed 7.5 pH. An appropriate volume of EE2 stock solution was added to a aqueous solution (500 mL or 1000 mL) of either unbuffered water or pH 7.5 phosphate buffer (0.01 M) to give a concentration of 6.75 pM (2 ppt) EE2. To this solution, an appropriate volume of either **1** or **2** stock solution was added to achieve a concentration of 80 nM. Degradation was then initiated by addition of an appropriate volume of H_2_O_2_ stock solution to give final concentrations of; 4.7, 47 or 470 μM (0.16, 1.6, 16 ppm). After 45 minutes, the reaction was quenched with catalase. In addition to the twelve reactions two ‘positive controls’ - 2 ppt EE2 spiked into pH 7.5 or unbuffered solutions, and a ‘negative control’ – double distilled water only, were also prepared (i.e. without the addition of **1**, **2** or H_2_O_2_). Each final solution was then concentrated with solid phase extraction (SPE) (Sep-Pak C18, Waters), and reconstituted in ethanol (0.5 mL or 1 mL to provide a 1000 fold concentration). Concentrated extracts were then assayed for estrogenic activity in a sensitive *in vitro* yeast estrogen screen (YES)^30^ a reporter gene assay incorporating a recombinant human estrogen receptor (LOD = 0.1 ng.L-1 or ppt).

### Degradation of EE2 in synthetic urine under controlled pH conditions

#### Materials

pH 7.4 synthetic urine was obtained from CST Technology, EE2 was obtained from Sigma Aldrich, sodium carbonate and hydrogen peroxide were obtained from Fisher, catalysts **1** and **2** were obtained as previously mentioned.

#### Method

Sample matrix was prepared by adding saturated sodium carbonate to synthetic urine until pH 8, 9 or 10 was achieved. EE2, catalyst, and hydrogen peroxide were added to give concentrations of 1-10 μM, 0.1-1 μM, and 0.6-6 mM respectively. EE2 concentrations were measured by a Waters 600E HPLC, 2996 PDA detector and 717 autosampler. Separation was performed on a Waters X-Terra RP8 150 × 4.6 column with gradient elution at the flow rate 1 mL/min. HPLC mobile phases includes MeCN and H_2_O. The injection volume was set at 20 μL. Wavelength 280 nm was selected to record the chromatogram.

### Degradation of EE2 and removal of associated biological effects *in vivo* – as measured by vitellogenin induction in male Fathead Minnows

The *in vivo* experiment was conducted to determine if EE2, and its associated biological effects could be significantly reduced by treatment under continuous flow-through conditions (as might be encountered at a wastewater treatment works). The fish used in this experiment are very sensitive to exogenous estrogens so a low but still ecotoxic EE2 concentration was used as well as a low (non-toxic) concentration of H_2_O_2_.

#### Test organism

Fathead minnows for the *in vivo* study were bred and reared at Brunel University’s U.K. Home Office licensed aquatic facility. Adult male fathead minnows (18 months old) were maintained in the tanks at 25 °C ± 1 °C with a photoperiod of 16:8 h light:dark. Fish were fed frozen brine shrimp twice a day and flake food once a day.

#### Experimental design

For the *in vivo* assay the four treatments were 1) negative control (no chemicals added), 2) positive control (EE2 only), 3) hydrogen peroxide treated EE2 (H_2_O_2_ and EE2) and 4) **2**/H_2_O_2_ treated EE2 (**2**, H_2_O_2_ and EE2) (Supplementary information Fig. S8). Each treatment regime consisted of two replicate tanks each with 8 male fathead minnows. Each chemical stock (EE2, H_2_O_2_ and **2**) was prepared and dosed separately so that the reaction only occurred in the mixing vessels (Supplementary information Fig. S8) prior to entering the fish tanks. Nominal concentrations (without reaction) in the mixing vessels were 2 ppt EE2, 80 nM **2**, 0.16 ppm H_2_O_2_. Water in the mixing vessels were continuously stirred by magnetic stirrer, retention/mixing time within the vessel were approximately 45 minutes before entering the fish tanks.

Fish (mean wet weight 3.9 ± 0.3 g) were placed into their tanks 1 week prior to chemical exposure to acclimatize. An additional 5 male fish were also included at this time to be sampled before chemical addition to provide baseline VTG data on day 0 of the assay. Dissolved oxygen (6.52 ± 0.4 mg/l), pH (8.33 ± 0.1) and water temperature (25.5 ± 0.2 °C) were recorded daily, as was the functioning of the dilution and dosing system. On days 6, 13 and 20 water samples were collected from the exposure tanks for estrogenic (EE2 and estrogenic activity) analysis. Water samples were collected in large 5 L silanized glass beakers and treated with catalase (Sigma-Aldrich) final concentration 0.2 ppm to prevent further catalyst reaction. Water samples were split between LC-MS/MS EE2 analysis and YES analysis^30^. Samples for YES were immediately concentrated with SPE (Sep-Pak C18, Waters) and reconstituted in ethanol for analysis. Samples for LC-MS/MS were fixed with an acidified copper nitrate (HCl/Cu(NO3)2) to prevent biological degradation. Analysis was conducted by LC-MS/MS with a limit of detection of 0.03 ppt. Technical replicates, blanks and EE2 spiked samples were also analysed (LC-MS/MS and YES).

On days 0 and 21 fish were sampled for plasma VTG. Fish were terminally anaesthetized with buffered MS-222 (Sigma-Aldrich) as approved by the U.K. Home Office (Animals (Scientific Procedures) Act 1986). Blood was collected using a heparinized hemocrit tube, the blood was centrifuged at 7,000 g for 5 min at 4 °C and the plasma collected and stored at -80 °C until analysis. Fish fork length and wet weight were recorded for each fish; condition factor was calculated as; (fish weight/fork length^3^) x100. Plasma VTG concentrations were measured via a quantitative enzyme-linked immunosorbent assay (ELISA) VGT kit designed specifically for fathead minnows (Biosense Laboratories, Norway). Plasma samples were diluted 1:50, 1:5,000, and 1:500,000 and assayed in duplicate according to the manufactures protocol.

#### Statistical analysis

Data (fish metrics and EE2 concentrations) were tested for homogeneity of variance. Parametric data was further analyzed using oneway ANOVA (followed by Least significant difference (LSD) post hoc analysis). Non parametric data was analyzed using Kruskal-Wallis Test (followed by Mann-Whitney U test post hoc analysis). EE2 chemical analysis results reported as <0.03 ppt EE2 (i.e. lower than detection limit (LOD)) were treated as having half LOD (i.e. 0.015 ppt EE2) for use in calculations of averages, standard error and statistical analysis. Data on biomarker responses were compared using oneway ANOVA followed by Tukey’s *post hoc* test (VTG data were log10 transformed prior to analysis). For EE2 and VTG concentrations independent T-tests were also used to assess if any significant differences occurred between tanks of the same treatment regime (tank effects). Statistical analysis was conducted using SPSS version 18. Differences were considered significant at p ≤ 0.05.

### Degradation of estrogens in Activated Sludge Process (ASP) effluent

The wastewater effluent experiments were conducted to assess the efficacy of treatment to remove steroid estrogens under a number of reaction conditions including; varying pH (7-10), H_2_O_2_ concentration (10-150 ppm) and retention time (15-45 mins). These parameters and their possible cost implications are important if TAML/H_2_O_2_ treatment is to be considered as an effective wastewater treatment technology.

#### ASP Effluent Study

Effluent was received from activated sludge sewage treatment plants in the UK. Samples were divided into containers of 2.5 litre volume and stored at room temperature. For samples at higher pH, the pH was adjusted with sodium hydroxide. Catalyst **1** and H_2_O_2_ (10, 20, 50, 100 or 150 ppm) were added to each sample and allowed to react for the designated amount of time (15, 30 or 45 mins). The reaction was quenched by addition of a reducing agent (ascorbic acid) to stop the oxidation reaction after a pre-determined time. An aliquot (2.5 litre) of the post reaction sample was transferred to a glass container, which was preserved with 3% v/v hydrochloric acid and 0.25% copper (II) nitrate. Samples were extracted using styrene-divinylbenzene solid phase extraction (SPE) cartridges and cleaned up using aminopropyl solid phase cartridges followed by gel permeation chromatography and analysed using LC-MS/MS to determine the percent removal of the estrogens studied.

## Additional Information

**How to cite this article**: Mills, M. R. *et al*. Removal of ecotoxicity of 17a-ethinylestradiol using TAML/peroxide water treatment. *Sci. Rep.* **5**, 10511; doi: 10.1038/srep10511 (2015).

## Supplementary Material

Supplementary Information

## Figures and Tables

**Figure 1:**
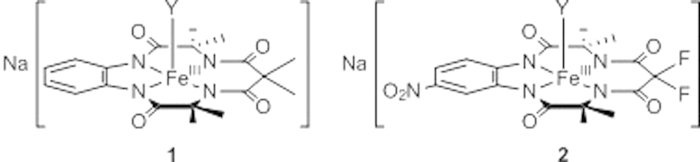
TAML activators **1** and **2** used in this study; Y is typically water.
