# Supplementary material for: Removal of ecotoxicity of 17α-ethinylestradiol using TAML/peroxide water treatment
Source: Sci Rep. 2015 Jun 12;5:10511. doi: 10.1038/srep10511 (PMC4464256; doi:10.1038/srep10511)
Supplement: Supplementary Information [file srep10511-s1.doc]

Supplementary Information

Removal of ecotoxicity of 17α-ethinylestradiol using TAML/peroxide water treatment

Matthew R. Mills^1^, Karla Arias-Salazar^1^, Alice Baynes^2^, Longzhu Q. Shen^1^,John Churchley^2^, Nicola Beresford^2^, Chakicherla Gayathri^1^, Roberto G. Gil^1^, Rakesh Kanda^2^, Susan Jobling2#* and Terrence J. Collins1#

1 Institute for Green Science and Department of Chemistry, Carnegie Mellon University, 4400 Fifth Avenue,

Pittsburgh PA 15213, USA

2 Institute of Environment, Health and Societies, Brunel University London, Uxbridge, Middlesex, UB8 3PH, United Kingdom.

**Supplementary Information Equation S1.
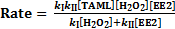
**

**
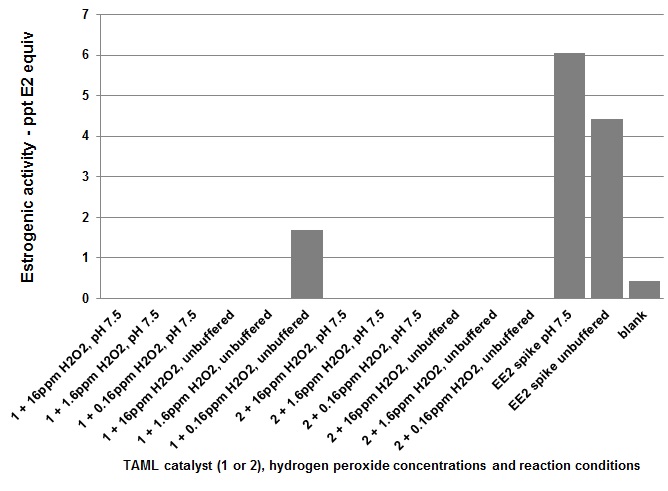
**

**Figure S1**. Comparison of twelve different reaction conditions for treatment of a low, environmentally relevant, concentration of EE2 (2 ppt, 6.75 pM) with 80 nM catalyst (**1** or **2**),with varying concentrations of peroxide (16, 1.6 and 0.16 ppm) and varying pH conditions (pH 7.5 or unbuffered). After 45 minutes of reaction time samples were quenched with catalase and concentrated 1000 fold prior to analysis. Estrogenic activity was measured using the yeast estrogen screen (YES) assays as described in the methods section. The pharmaceutical estrogen EE2 is approximately three times as potent in the YES assay as the natural steroid estrogen estradiol (E2) used as a reference standard in this assay.

**
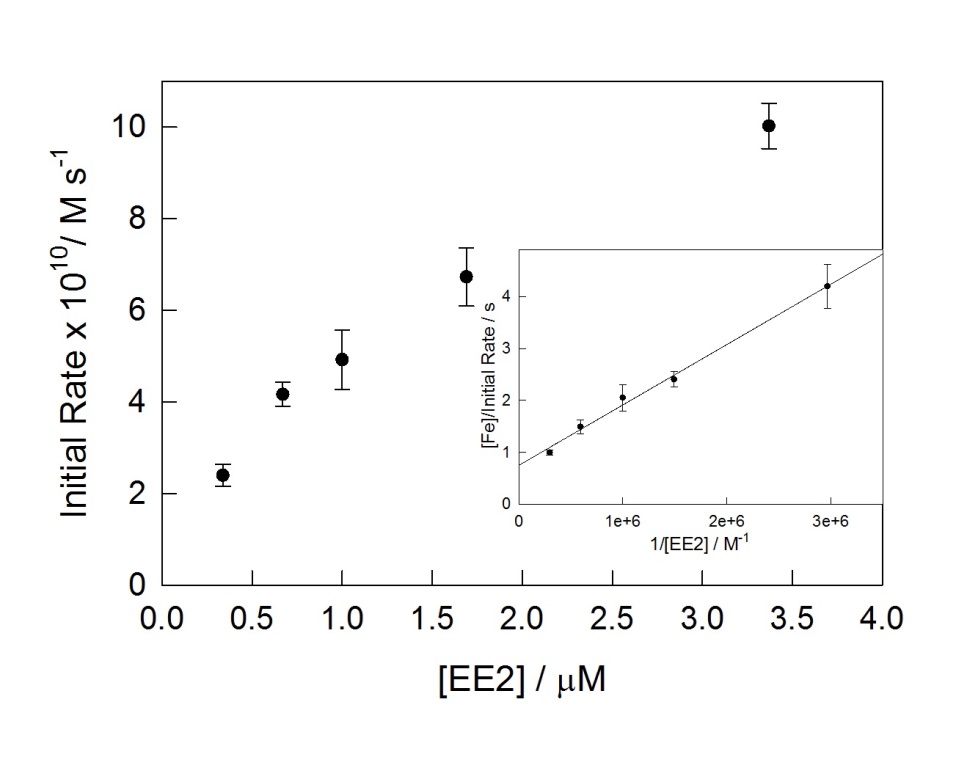
**

**Figure S2**. *A:* Plot of the hyperbolic dependence of the initial rate on EE2 concentration. All points are the average of at least three trials with the error bars indicating standard deviation. *B:* Plot obtained by graphing the catalyst concentration divided by the initial rate versus the inverse of EE2 concentration. The slope of the linear regression is 1/*k*II (*k*II is the rate constant for the reaction of the TAML reactive intermediate with the EE2) and the intercept is 1/*k*I[H2O2] (*k*I is the rate constant for the reaction of the Fe(III)-TAML catalyst with H2O2 to form the reactive intermediate). Conditions: 0.01 M Phosphate buffer, pH 7, [H2O2] = 0.01 M, [**1**] = 1.0x10^-9^ M. EE2 concentration monitored by HPLC as described in the methods section.

**
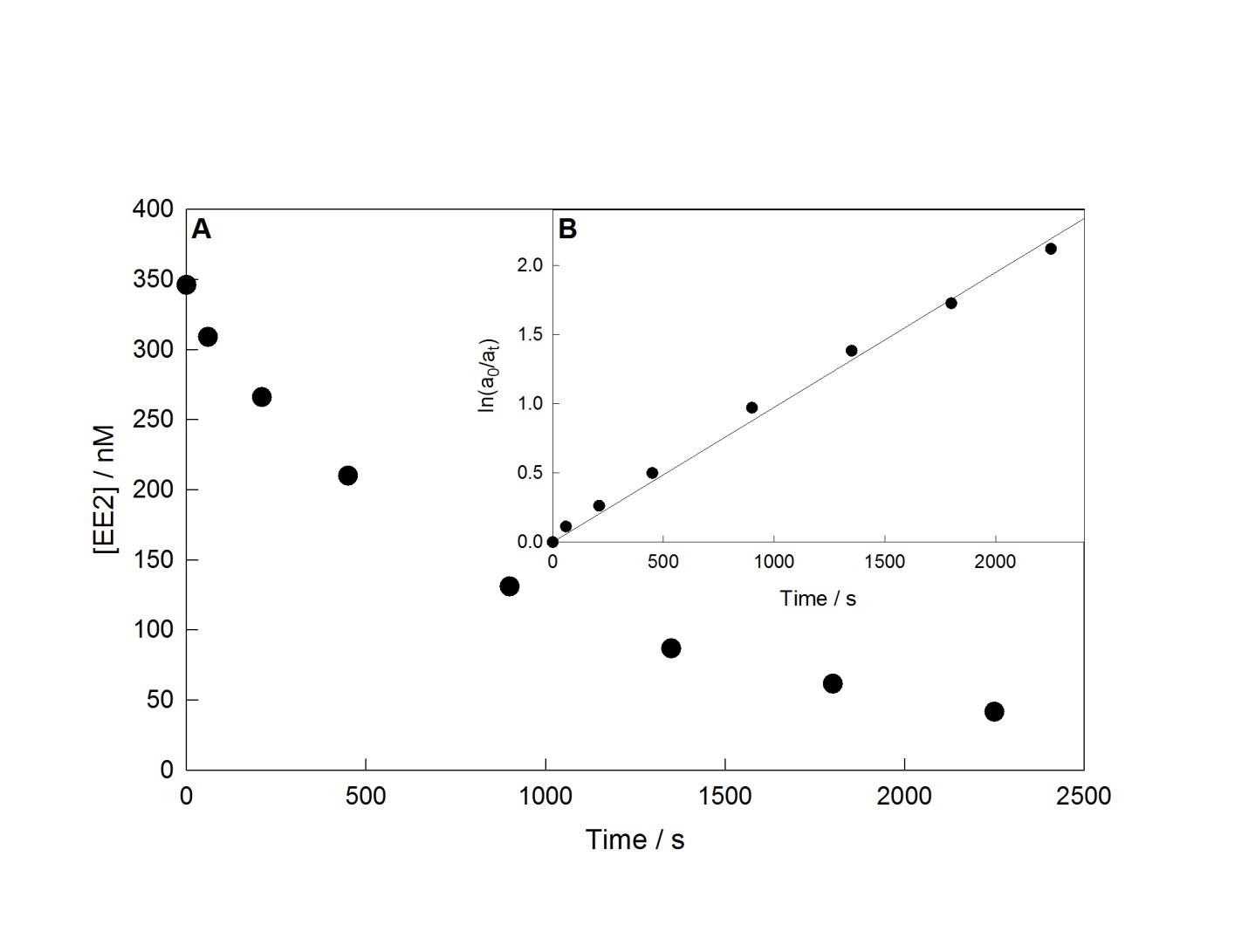
**

**Figure S3**. *A:* Exponential decay of EE2 under *k*II-limiting conditions, where kI[H2O2]>>kII[EE2] and the rate equation simplifies to rate = *k*II[EE2] (assuming [**1**] constant). Conditions: 0.01 M Phosphate buffer at pH 7, [H2O2] = 0.010 M, [**1**] = 1.0x10^-9^ M. *B*: Linearization of the data in plot A by plotting ln([EE2]initial/[EE2]t) vs. time. The slope of the linear regression gives *k*II[**1**], which yields a *k*II value of 9.4 +/- 0.2 x10^-5 M^-1^ s^-1^, verifying the value that was obtained from varying the peroxide concentration.

**
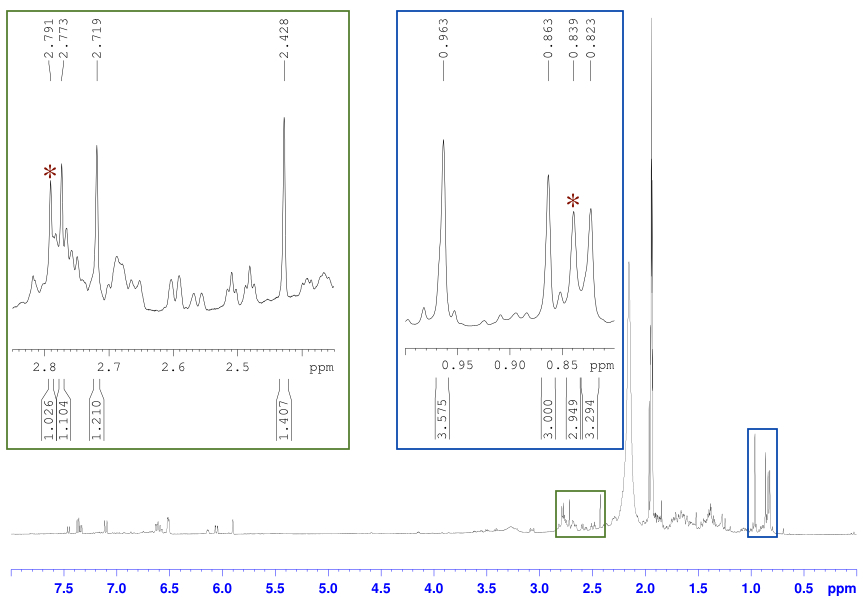
**

**Figure S4.** Full ^1^H NMR spectrum of the intermediate mixture that results from treating EE2 with Fe-TAML/H2O2. The insert in green corresponds to the terminal acetylene signals, and the insert in blue corresponds to the methyl group signals. The signals corresponding to **3** in the reaction mixture are marked with red asterisks. Data in Supplementary Information Figures 4-7 were obtained from 1L-scale reactions, followed by concentration with SPE and isolation with preparatory HPLC as described in the Experimental Section. Conditions: [EE2] = 3.37x10^-5^ M, [**1**] = 8.0x10^-8^ M, [H2O2] = 3.37x10^-4^M, 0.01 M pH 9 phosphate buffer, room temperature. Conditions: [EE2] = 3.37x10^-5^ M, [**1**] = 8.0x10^-8^ M, [H2O2] = 3.37x10^-4^ M, 0.01 M pH 9 phosphate buffer, room temperature.

**
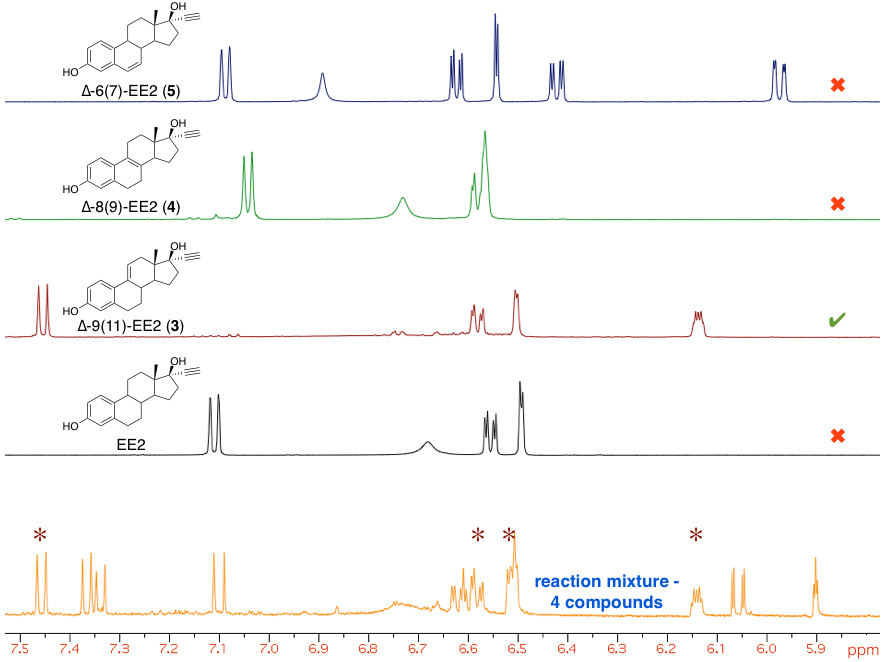
**

**Figure S5.** A comparison of the aromatic regions of the ^1^H NMR spectra of the reaction mixture (in orange), EE2 (in black) and possible oxidative degradation products **3** (in red), **4** (in green), and **5** (in blue). The signals corresponding to **3** in the reaction mixture are marked with red asterisks. For conditions see Supplementary Information Figure 4.

**
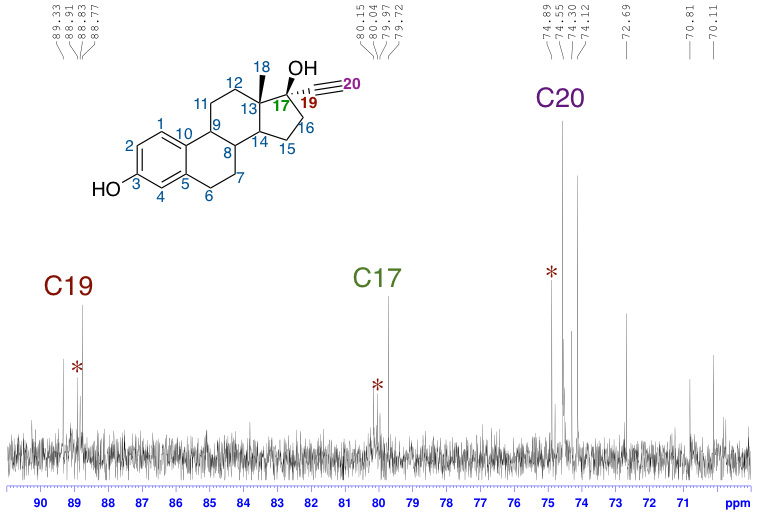
**

**Figure S6.** The 70 – 90 ppm region of the ^13^C NMR spectrum of the degradation intermediate mixture. The signals corresponding to intermediate **3** in the reaction mixture are marked with a red asterisk. For conditions see Supplementary Information Figure 4.

**
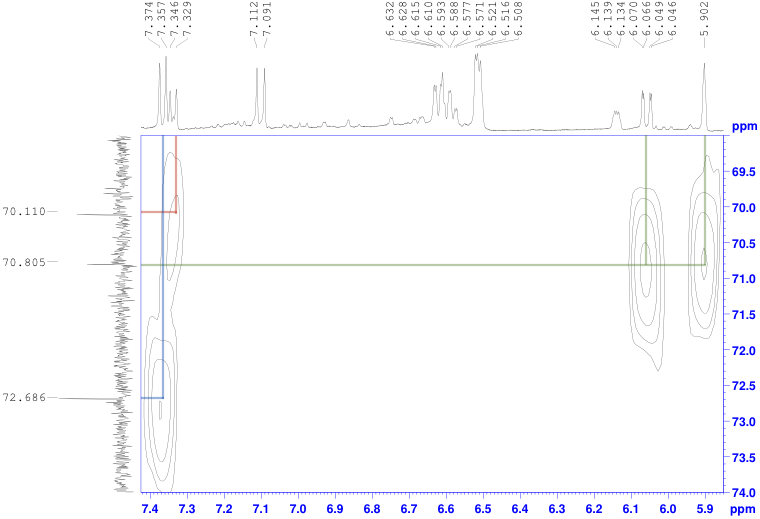
**

**Figure S7.** HMBC (^1^H and ^13^C) of the degradation intermediate mixture obtained from preparatory HPLC. The spectrum shown focuses on the correlations between the ^1^H aromatic region and the unassigned ^13^C peaks at ~70 ppm. This was used to verify the structures of the intermediates proposed. For conditions see Supplementary Information Figure 4.


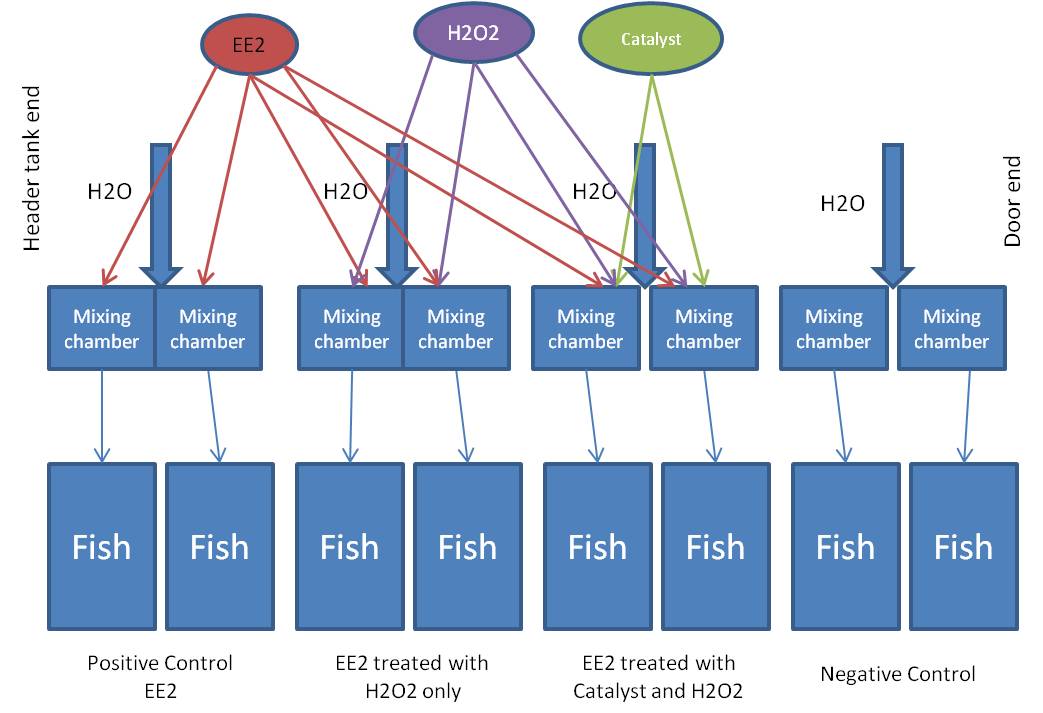


**Figure S8. Diagram of *in vivo* fathead minnow 21-day flow-through bioassay.**

The experimental set up consisted of 8 11L glass aquaria each fed with continuous flow of water. Individual chemical stock solutions (EE2 in 0.1% ethanol in double distilled water (ddH_2_O), 80μM 2 in ddH_2_O and H_2_O_2_ in ddH_2_O) and filtered dechlorinated water were delivered to mixing chambers (2L aspirator bottles, working volume 1.5L) at a rate of 0.033ml/min (Watson Marlow multichannel peristaltic pump) and 33.33 ml/min (gravity fed flow meter) respectively. Therefore, giving a thousand-fold dilution and nominal concentrations (without reaction) in the mixing vessels of 2 ppt EE2, 80 nM 2, 0.16 ppm H_2_O_2_. Chemical stock solutions (EE2, H2O2 and **2**) were prepared and dosed separately so that the reactions commenced in the mixing vessels. Fish were exposed to the mixture(s) after a reaction contact time of approximately 45 minutes.
